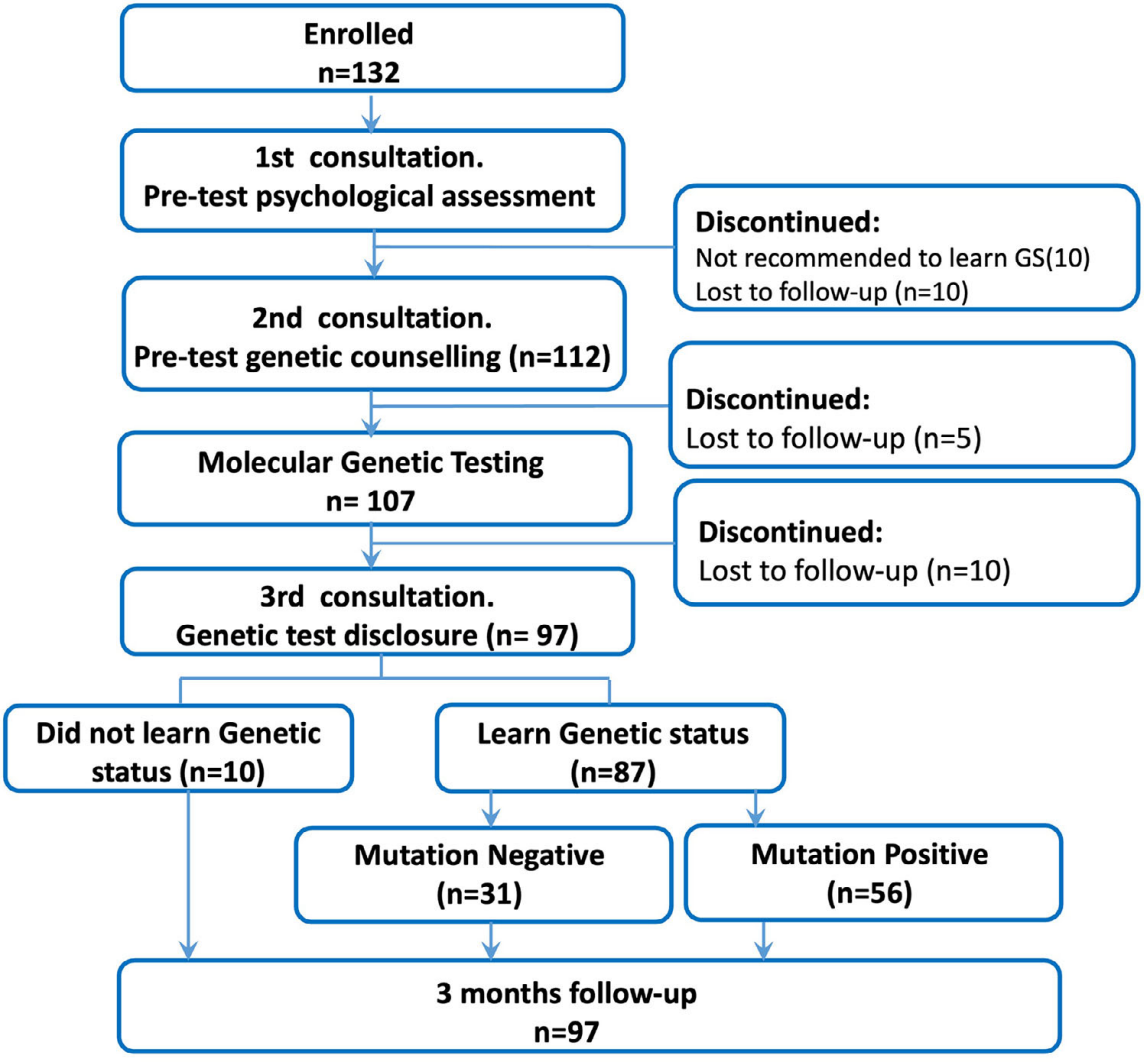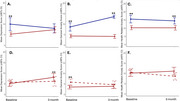# Impact of genetic counseling and testing in individuals at high risk for Alzheimer ´s Disease from Latin America

**DOI:** 10.1002/alz70857_099318

**Published:** 2025-12-24

**Authors:** Pablo Miguel Bagnati, David Aguillon, Marisol Londoño Castaño, Maria L Fernandez, Beatriz E Mora‐Henao, Patricio Chrem, Luz E Varela Londoño, Juan D Barbaran Manco, Yudy M Leon‐Varela, Ezequiel Ignacio Surace, Claudia Madrigal, Juan Pablo Picasso, Claudia Ramos, Carlos M Restrepo‐Fernández, Gabriela Vigo, Laura Ramirez Aguilar, Gabriel Vargas, Mauricio Arcos Burgos, Erika Mariana Longoria, Ellen Ziegemeier, Eric McDade, Randall J. Bateman, Ricardo Allegri, Francisco Lopera, Jorge J. Llibre‐Guerra

**Affiliations:** ^1^ Fleni, Buenos Aires, Buenos Aires, Argentina; ^2^ Neurosciences Group of Antioquia, University of Antioquia, Medellín, Colombia; ^3^ Grupo de Neurociencias de Antioquia, Facultad de Medicina, Universidad de Antioquia, Medellín, Antioquia, Colombia; ^4^ Fleni, Buenos Aires, Argentina; ^5^ Fundación Médica de Enfermedades Raras, Medellín, Colombia; ^6^ Grupo de Neurociencias de Antioquia, Facultad de Medicina, Universidad de Antioquia, Medellín, Colombia; ^7^ Neurosciences Group of Antioquia, University of Antioquia, Medellín, Colombia; ^8^ FLENI, CABA, Buenos Aires, Argentina; ^9^ Hospital San Bernardo de Salta, Salta, Argentina; ^10^ Univerdidad de Antoquia, Medellin, Colombia; ^11^ g. Cognitive Neurosciences Lab, National Institute of Neurology and Neurosurgery, Mexico, Mexico, Mexico; ^12^ Washington University in St. Louis School of Medicine, St. Louis, MO, USA; ^13^ Department of Neurology, Washington University School of Medicine, St. Louis, MO, USA; ^14^ Washington University School of Medicine, St. Louis, MO, USA; ^15^ Grupo de Neurociencias de Antioquia, Universidad de Antioquia, Medellin, Colombia

## Abstract

**Background:**

This study evaluates a tailored genetic counseling and testing (GTC) protocol for families at risk of Autosomal Dominant Alzheimer ´s Disease (ADAD) in Latin America, focusing on the essential cultural and regional adaptations. Several factors may influence the decision of whether family members decide to learn genetic status. Although the main drivers influencing the decisions to seek genetic testing have been widely studied in High‐Income Counties (HIC), these questions remain relatively unknown in Low and Middle‐Income countries (LMICs) such as those in Latin America (LatAm).

**Method:**

The primary aim of this study was to investigate the psychosocial impact of genetic testing in asymptomatic individuals from families with ADAD. We conducted a non randomized, controlled trial among ADAD families in Colombia and Argentina. The primary outcome included change from baseline in depression and general anxiety in the test group relative to the control group. Participants were categorized based on their decision to learn their genetic status, with further comparisons between mutation‐positive versus mutation‐negative individuals within those informed. Psychological impacts were measuring using validated scales for depression and anxiety in the group relative to the control group.

**Result:**

Of the 122 eligible participants, 97 completed the GTC protocol; 87 opted to learn their genetic status. There were no clinically significant differences in psychological distress between those who learned their status and those who did not, nor between mutation‐positive and mutation‐negative individuals. Mutation‐positive carriers who learned their genetic status experienced a statistically significant increase in depression scores but remained below the cut‐off point considered indicative of clinically significant depression.

**Conclusion:**

Our primary finding is showed no clinically meaningful differences in distress‐related outcomes between individuals learning their genetic status relative to those who didn’t. Our findings confirm that genetic testing is well‐tolerated when using a protocol that provides screening, education, counseling, and follow‐up sessions. In addition, we provide preliminary insights into the psychological impact associated with learning one's genetic status in familial AD, contributing significantly to the field of medical genetics in the region.